# Phenotype Design Space Provides a Mechanistic Framework Relating Molecular Parameters to Phenotype Diversity Available for Selection

**DOI:** 10.1007/s00239-023-10127-y

**Published:** 2023-08-25

**Authors:** Michael A. Savageau

**Affiliations:** 1 Department of Microbiology & Molecular Genetics, University of California, 228 Briggs, Davis, CA 95616 USA; 2 Department of Biomedical Engineering, University of California, One Shields Avenue, Davis, CA 95616 USA

**Keywords:** Biochemical systems theory, Circadian clock circuitry, Theoretical population genetics, Fisher’s geometric model, Constructive neutral evolution, Evolutionary dynamics

## Abstract

Two long-standing challenges in theoretical population genetics and evolution are predicting the distribution of phenotype diversity generated by mutation and available for selection, and determining the interaction of mutation, selection and drift to characterize evolutionary equilibria and dynamics. More fundamental for enabling such predictions is the current inability to causally link genotype to phenotype. There are three major mechanistic mappings required for such a linking – genetic sequence to kinetic parameters of the molecular processes, kinetic parameters to biochemical system phenotypes, and biochemical phenotypes to organismal phenotypes. This article introduces a theoretical framework, the Phenotype Design Space (PDS) framework, for addressing these challenges by focusing on the mapping of kinetic parameters to biochemical system phenotypes. It provides a quantitative theory whose key features include (1) a mathematically rigorous definition of phenotype based on biochemical kinetics, (2) enumeration of the full phenotypic repertoire, and (3) functional characterization of each phenotype independent of its context-dependent selection or fitness contributions. This framework is built on Design Space methods that relate system phenotypes to genetically determined parameters and environmentally determined variables. It also has the potential to automate prediction of phenotype-specific mutation rate constants and equilibrium distributions of phenotype diversity in microbial populations undergoing steady-state exponential growth, which provides an ideal reference to which more realistic cases can be compared. Although the framework is quite general and flexible, the details will undoubtedly differ for different functions, organisms and contexts. Here a hypothetical case study involving a small molecular system, a primordial circadian clock, is used to introduce this framework and to illustrate its use in a particular case. The framework is built on fundamental biochemical kinetics. Thus, the foundation is based on linear algebra and reasonable physical assumptions, which provide numerous opportunities for experimental testing and further elaboration to deal with complex multicellular organisms that are currently beyond its scope. The discussion provides a comparison of results from the PDS framework with those from other approaches in theoretical population genetics.

**Supplementary Information:**

The online version contains supplementary material available at 10.1007/s00239-023-10127-y.

## Introduction

The concept of evolution is easily stated and understood: Mutation generates diversity of phenotypes and selection favors those with the greatest heritable fitness. However, there are many complex and inter-related issues that must be addressed to achieve a deeper understanding. Two prominent examples that continue to be fundamental challenges are (1) determining the *distribution of phenotype diversity*, which offers opportunities for innovation (Charlesworth 1996; Bataillon & Bailey 2014) and (2) determining the interaction of *mutation, selection, drift and population structure* to determine equilibria and the dynamics of evolution (Gillespie 2004; Orr 2005; Wakeley 2005).

While advances in genome sequencing technology (Metzker 2010) can provide distributions of the numbers and types of changes in DNA, determining the distribution of the resulting phenotypes and their fitness characteristics (determinants of total fitness) in natural populations is difficult in the extreme (Charlesworth 1996). The true number of phenotypes and fitness characteristics in the population is typically unknown and any observed distribution of total fitness (e.g., growth rate of bacteria) is skewed by what can be observed in the field, and measured or generated experimentally in the laboratory (Gallet et al. 2012; Robert et al. 2018; Bondel et al. 2019; Lebeuf-Taylor, et al., 2019). With large data sets, correlations can be established between genome changes and fitness changes in a given environment. However, at a fundamental level there are many fitness components based on function that remain to be identified and characterized and many unsolved mappings that prevent a predictive, causal linking of mutations in DNA, properties of molecular components, integrated system function, phenotypic repertoire, and fitness. In short, there is little relevant theory for guidance.

There is a rich field of theoretical population genetics developed over more than a century that addresses the interaction of *mutation, selection, drift and population structure* (Gillespie 2004; Orr 2005; Wakeley 2005). However, aside from the simpler cases of one-to-one mapping between gene and phenotypic function, an appropriate theoretical framework is lacking to pose and answer questions for the more complex cases that involve mappings between many genes and many functional contributions to phenotypes.

In reviewing the genetic theory of adaptation, Orr (2005) examined “the reasons a mature [mathematical] theory has been slow to develop and the prospects and problems facing current theory” and concluded that although recent models “seem to successfully explain certain qualitative patterns […] future work must determine whether present theory can explain the genetic data quantitatively”. Experimental evolution studies have shown that mutations in a single gene affecting a specific enzyme can lead to a marked change in organismal fitness (Barrick & Lenski 2013; Gresham & Jong 2015). Although the results might be explained qualitatively, without an adequate systems theory these explanations cannot provide a rigorous, quantitative, causal understanding of the complex underlying events.

**Fig. 1:**
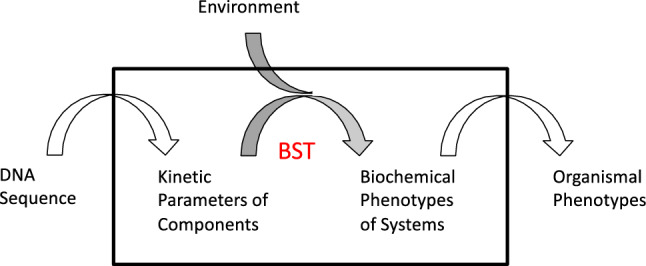
Three major mappings between genotype and phenotype. The mapping from genetically determined kinetic parameters and environmentally determined variables to biochemical system phenotypes is the subject of biochemical systems theory (BST) that is utilized for this work

Can knowledge of molecular systems tell us anything about the distribution of mutant phenotypes and their evolution? A large part of the problem in relating molecular mechanisms to phenotype distributions and evolution is the inability to relate the genotype and environment to the phenotype exhibited by a biological system, which is one of the ‘Grand Challenges’ in biology (Brenner 2000). The causal linking of genotype to phenotype involves at least three essential mappings (Fig. 1).

First is the mapping from the *digital values* of the genome sequence to the *analogue values* of the kinetic parameters that characterize the underlying molecular processes. Second is the mapping from the *kinetic parameters* of the individual component processes to the quantitative *biochemical phenotypes* of the integrated cellular system. Third is the mapping from the *biochemical* (endo-) *phenotypes* to the *organismal (exo-) phenotypes*, including observables such as growth rate, taxis and adhesion. The first of these mappings deals with protein structure function relationships, which relate DNA sequence to properties of the encoded protein. Recent success in solving the protein folding problem (Callaway 2020) bodes well for the eventual ability to predict kinetic parameters. The second mapping is the focus of Biochemical Systems Theory (Savageau 1971; 2009; Voit 2000; 2013), which in the past decade has provided a novel system deconstruction that maps genetically determined parameters and environmentally determined variables to biochemical phenotypes. The result is a highly structured partitioning of parameter space that is defined as the *System Design Space when referring to dynamics of the underlying molecular system* (Savageau et al. 2009) and as the *Phenotype Design Space (PDS) when referring to dynamics of the evolving population*. A Design Space Toolbox (DST3) is available with numerous tools that automate the analysis (Valderrama-Gómez et al. 2020). The third of these mappings is perhaps the most difficult, in any but the simplest cases of one-gene one-protein one-phenotype, due to the large number of genes and phenotypes with many-to-many interactions that currently can only be characterized by large data sets and statistical correlations (McCarthy et al. 2008; Greenbury et al. 2016).

**Fig. 2:**
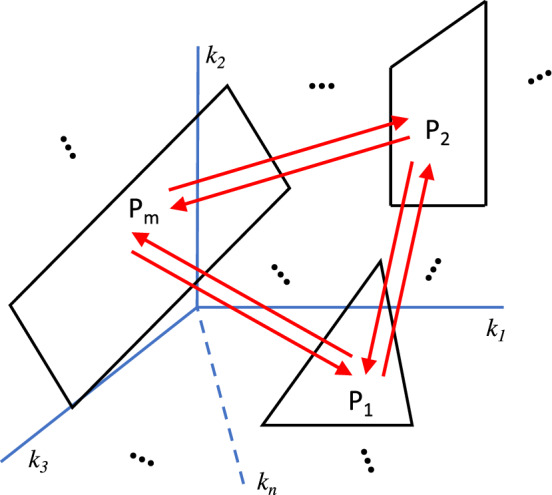
Schematic representation of the overall strategy. This figure depicts a two-dimensional slice of the *n*-dimensional space of molecular parameters (*k*_*1*_*, k*_*2*_*, …, k*_*n*_) but shows only three planes of the *m* volumes that fill the entire space. Specific volumes (black polytopes) in the *n*-dimensional space correspond to qualitatively distinct system phenotypes (P_1_, P_2_, …, P_m_). Transition probabilities among phenotypes due to mutation (red arrows) are determined by mathematically defined volumes of phenotypes and distances and biases between phenotypes

If one could enumerate the full repertoire of phenotypic functions that could be exhibited by a given biological system and know the rates of transition among them in the population undergoing mutational exchange, then one would have a deep understanding of the functional basis for phenotypic diversity and plasticity available for selection to act upon. Here we address these issues in five parts. A schematic of the overall strategy is depicted in Fig. 2. The technical methods involve reasonable physical assumptions derived from fundamental biochemical kinetics as well as linear algebra, as shown in the following sections and **Supplemental Information (SI).**

The first part introduces a small molecular system, a hypothetical primordial precursor to a circadian clock that provides a context and an aid to understanding the approach. This system is specifically selected for this purpose because it provides a hypothesis-motivated example with unknown parameter values. Any real system will initially have many unknowns and involve the formulation of, and discrimination among, many hypotheses that require experimental testing; the Design Space approach has advantages to offer specifically at this stage of an investigation (Lomnitz & Savageau 2016a). The second part extends the Design Space concepts **(SI: Sect. 1)** to the PDS framework by applying the phenotype-centric strategy to predict phenotype-specific mutation rate constants. This involves formulating phenotype-specific mutation rates based on transition probabilities between biochemical phenotypes. These rate constants are then used to formulate population dynamic equations for predicting equilibrium distributions of phenotype diversity under non-selecting and selecting conditions. The third part presents results for the population genetic model. The fourth part discusses specific predictions in the light of experimental challenges for their testing. The fifth part compares theoretical results provided by the PDS framework with those provided by other approaches.

## Putative Primordial Circadian Clock

The molecular system, treated as a case study here, is related to the positive–negative feedback module found at the core of nearly all circadian clocks (Bell-Pedersen et al. 2005; Hardin 2011; Cohen & Golden 2015; Nohales & Kay 2016; Papazyan et al. 2016; Creux & Harmer 2020) and several synthetic oscillator designs (Atkinson et al. 2003; Stricker et al. 2008; Tigges et al. 2009; Lomnitz & Savageau 2014). In the transcription-translation oscillators, this module consists of a positive transcription factor that activates its own synthesis as well as synthesis of a negative transcription factor, which in turn represses synthesis of the positive transcription factor. The module originally identified in *Drosophila* is elaborated upon in animals (Preitner etal., 2002) and plants (Creux & Harmer 2020) with numerous variations on the theme, including diverse input stimuli that modulate expression of one or both factors (Balsalobre, et al. 2000; O’Neill & Reddy 2012) and rich output interactions with nearly all cellular functions (Creux & Harmer 2020).

In the cyanobacterial clock, the transcription-translation mechanism is a minor player whereas a posttranslational oscillator mechanism with different positive and negative interactions plays the dominant role (Cohen & Golden 2015). When growing exponentially in a normal diurnal light cycle, phenotypes without the oscillatory characteristic are at a selective disadvantage when compared to the wild-type (oscillatory phenotype); however, they exhibit no measurable disadvantage when grown under the non-selecting condition (constant light), as determined by growth competition between mutants and wild-type in an otherwise isogenic background (Ouyang et al. 1998).

Roenneberg & Merrow (2002) and many others have speculated that the robust limit cycle, or sustained oscillation, exhibited by circadian clocks in modern organisms is unlikely to have arisen full blown. Some of the coordinating functions could have been provided by a simpler core module having a damped oscillation with a frequency that resonates to and becomes synchronized with the diurnal cycle. Indeed, such damped oscillations have been experimentally observed in strains of cyanobacteria: namely, clock mutants of *Synechococcus* (Ouyang et al. 1998; Kawamoto et al. 2020) and marine *Prochlorococcus marinus* (Holtzendorff et al. 2008).

**Fig. 3:**
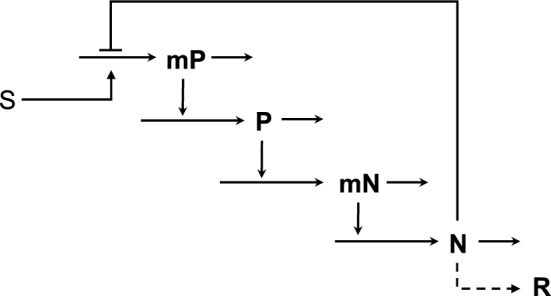
Common genetic module for the putative precursor of the modern core mechanism of nearly all circadian clocks. Positive (P) and negative (N) transcription factor proteins and the corresponding mRNAs (mP and mN). Environmental input stimulus (S) and biochemical output response (R) are suggestive only, other targets and coordinating signals could be considered. See also SI: Section S2

Because mutants without the oscillatory characteristic exhibit no measurable disadvantage when grown under the non-selecting conditions, this suggests a number of scenarios by which the elements of a primordial clock could evolve according to nearly neutral theory (Kimura 1983; Ohta 1992). The two-transcription factor bindings, which is the minimum number needed to form the necessary negative feedback loop, could arise in either order. Establishing links to the input from the environmental signal and to the output to cellular metabolism, also could arise in either order. The combination of these events would form the basic architecture of the model in Fig. 3. However, this would still be insufficient to generate damped oscillations; this would require the evolution of sufficient cooperativity in the two bindings (e.g., by dimerization of each protein), which also could arise in either order. Lacking any one of these events, the primordial clock would still experience no measurable disadvantage. Only when all the events have been established would the oscillatory primordial clock have a selective advantage under selecting conditions and its non-oscillatory mutants be at a disadvantage.

These novel elements might result from duplication and repurposing one of the duplicates (Stoltzfus 1999), although a more effective scenario for the acquisition of new functions has been predicted based on the Demand Theory of gene regulation (Savageau 1989). The prediction consists of three elements (1) functions in low demand are predicted to be under negative control, (2) in any given context there are a large number of such quiescent functions and a loss-of-function mutation in the regulator for any one will produce a greatly amplified increase in the expression of the corresponding function, and (3) since most functions have a range of promiscuous activity for functions other than the native (Khersonsky & Tawfin, 2010; Rueda et al. 2019), even a small percentage of such activity will result in a substantial increase in activity for the realization of the newly acquired function. Experimental evidence confirms these predictions of the Demand Theory: phenotypes determined by mechanisms subject to negative regulation frequently arise by loss-of-function mutations in their negative regulatory components to yield newly acquired functions (McDonald et al. 2009; Tenaillon et al. 2012; Lind et al. 2015, 2019; Fraebel et al. 2017).

For our purposes here, consider the primordial mechanisms to involve only the negative feedback loop at the core of modern transcription-translation mechanisms, as shown schematically in Fig. 3. The equations used to model this putative primordial clock (**SI: Section 2**) are based on the foundation of fundamental biochemical kinetics. Such models have broad general applicability, as the vast majority of biochemical models are of this type (Chelliah et al. 2013). These equations, recast as an equivalent GMA-system of equations (**SI: Section S3**), become a set of differential–algebraic equations in the syntax of the Design Space Toolbox (Lomnitz & Savageau 2016b).

Three assumptions simplify the presentation. (1) The precursor is likely to involve a minimal number of processes and a minimal degree of cooperativity in the interactions. The two-transcription factor model involves at least four processes and two cooperative DNA interactions. For it to generate a damped oscillatory response, the system must be near the threshold of instability, which requires a value of loop cooperativity [(n*p) in the **Eqn.** (**S1**) to **Eqn.** (**S4**)] equal to 4 for a system with four temporally dominant stages (Savageau 1975; Thron 1991). Let the cooperativity parameters each have the minimum value *n* = *p* = 2. [Kawamoto et al. (2020) considered a three-factor model for *Synechococcus*; but it requires a much higher degree of cooperativity, *n* > 8 as shown in Savageau (1975).] (2) To provide the most challenging shape for testing different methods of volume calculation (**SI: Section S5, Fig. S2**), we select values for the two parameters (capacity for regulation for the two transcripts) with the potential to break the symmetry such that a skewed volume is generated for the phenotype with an oscillatory characteristic. (3) To aid visualization of the results we focus on a two-dimensional slice through the Design Space with the two binding constants displayed on the vertical and horizontal axes. This choice provides a representative view of the invariant for the Design Space of this system (**SI: Section S4, Fig. S1A**).

The Design Space Toolbox 3 (DST3, Valderrama-Gómez et al. 2020) is used to enumerate the repertoire of phenotypes *without assuming values for any of the model’s kinetic parameters*, and the results demonstrate a maximum of nine possible phenotypes. These are listed in Table 1, along with the properties of their eigenvalues when *n* = *p* = 2. Each sequential pair of integers in the phenotype signature identifies the specific positive and negative terms in the corresponding GMA equation that are instrumental in defining the phenotype. A comparison of the phenotype signatures with the GMA equations (**SI: Section S3)** identifies the specific S-system equation for each phenotype. For example, the phenotype signature for phenotype #7 (11 11 21 11 21 11) indicates that the first positive and first negative terms of the first GMA equation (**Eqn. S5**) are dominant, the first positive and first negative terms of the second GMA equation (**Eqn. S6**) are dominant, the second positive and first negative terms of the third GMA equation (**Eqn. S7**) are dominant, etc. When the resulting auxiliary variables DP and DN are substituted into the differential equations and these are converted back to their biochemical kinetic form, the corresponding S-system for phenotype #7 is given by **Eqns. **(1–4).

1 $$ \frac{dmP}{{dt}} = \,\alpha_{mPmax} \left( {\frac{N}{{K_{N} }}} \right)^{ - n} - \beta_{mP} mP $$

2 $$ \frac{dP}{{dt}} = \alpha_{P} mP - \beta_{P} P $$

3 $$ \frac{dmN}{{dt}} = \alpha_{mNmax} \left( {\frac{P}{{K_{P} }}} \right)^{p} - \beta_{mN} mN $$

4 $$ \frac{dN}{{dt}} = \alpha_{N} mN - \beta_{N} N $$

**Table 1:** Phenotypic repertoire for the model in Fig. 3

Phenotype Number	Phenotype Signature	Eigenvalues with Positive real part	Complex Conjugate Eigenvalues
1	11 11 11 11 11 11	0	–
3	11 11 11 11 21 11	0	–
5	11 11 21 11 11 11	0	–
6	11 11 21 11 11 21	0	–
7	11 11 21 11 21 11	0	+
8	11 11 21 11 21 21	0	–
11	21 11 11 11 21 11	0	–
15	21 11 21 11 21 11	0	–
16	21 11 21 11 21 21	0	–

Results determined using only **Eqns.** (**S5** to **S12**)

All phenotypes are stable (no eigenvalues with positive real part) with no complex conjugate eigenvalues (no possibility of oscillations), except for phenotype #7; thus, only phenotype #7 has the potential to initiate damped oscillatory behavior. This is the only phenotype for which both transcription factors are operating within their regulatable region.

It should be emphasized that the enumeration of the full phenotypic repertoire by DST3 is accomplished *without having to specify values for any of the thermodynamic or kinetic parameters*. By specifying the stoichiometry for binding repressor and activator as *n* = 2 and *p* = 2, DST3 automatically predicts scaled values for all 12 thermodynamic and kinetic parameter values of the system, identifies the region in Design Space for the realization of phenotype #7, the phenotype of interest here, as well as the steady-state values of the four dynamic variables. By choosing the simplest scaling, generating a skewed volume for phenotype #7, and shifting the entire Design Space to center the visualization on phenotype #7 (**SI: Section S4, Fig. S1A**), we predict values for the 12 parameters and the steady-state values for the four dynamic variables as shown in Table 2.

**Table 2:** Scaled values for the parameters and steady-state concentrations automatically determined at the centroid for phenotype #7 (11 11 21 11 21 11). The behavior of the model is determined by these scaled parameter values

KN	0.316	aN	1.00
KP	1.78	aP	0.01
amNmax	10.0	bmN	1.00
amNmin	1.00	bmP	1.00
amPmax	10,000	bN	1.00
amPmin	1.00	bP	1.00

If necessary, twelve experimental measurements (e.g., maximum expression, minimum expression and lifetime of each mRNA and protein) are sufficient to determine the actual parameter values. However, as can be seen in **Eq. **(5), the methods involve *differences* in log space so the scale factors cancel out and thus there is no effect on the qualitative or quantitative results. Predicted normalized steady-state values: mP = 100.0; P = 1.0; mN = 3.16; N = 3.16

**Fig. 4:**
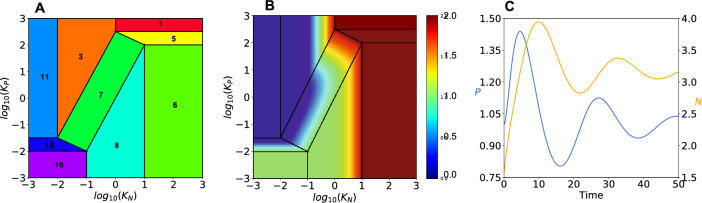
Predicted phenotype characteristics in Design Space. **A** Visualization of phenotype regions. Region of oscillatory phenotype #7 is the central rectangular shape. **B** Steady-state concentration of total protein (N + P) plotted log10 as a heat map on the z-axis. **C** Validated oscillatory behavior for phenotype #7. Concentrations of activator P (left y-axis, Blue) and repressor N (right y-axis, Gold) as a function of time scaled by a factor of 1/3. Initial conditions are: mP = 100; P = 1.0; mN = 3.16; N = 1.58. Figures generated with the following parameter values: KN = 0.316; KP = 1.78; aN = 1.0; aP = 0.01; amNmax = 10.0; amNmin = 1.0; amPmax = 10,000.0; amPmin = 1.0; bN = 1.0; bP = 1.0; bmN = 1.0; bmP = 1.0; Kinetic order(s): n = 2, p = 2; (The parametric constraints amPmax > amPmin and amNmax > amNmin are automatically satisfied by this parameterization of the model.)

Although variation in all 12 parameters could be explored, we focus on the two equilibrium dissociation constants *K*_*P*_ and *K*_*N*_, which will be allowed to vary because of mutation. This simplification reduces the dimensions of the Design Space for ease in visualizing the results while providing an accurate representation of the underlying Design Space invariant. The size of the regions in Design Space occupied by each of the phenotypes (Fig. 4A) then can be determined by a vertex enumeration method (Avis 2000; Barber et al. 1996). These methods work well for small systems and other methods are available for large systems (**SI: Section S5, Figs. S3 & S4**).

## Phenotype-Specific Mutation Rate Constants

The Design Space enables a novel ‘phenotype-centric’ modeling strategy that is radically different from the conventional ‘simulation-centric’ approach (Valderrama-Gómez et al. 2018). A summary of Design Space concepts is provided (**SI: Section S1)** to facilitate understanding of the phenotype-centric strategy used to predict phenotype-specific mutation rate constants in the PDS framework.

The mechanistic PDS framework proposed here requires new concepts and methods; the reader is directed to **Supplemental Information** where these are fully developed. It involves analysis at two different levels of organization that must be clearly distinguished: dynamics at the intracellular level of biochemistry and dynamics at the extracellular level of population numbers. The former is well developed elsewhere and used as part of the analysis; however, the latter is new and is the focus here. Four factors in Design Space contribute to the probability of transition between phenotypes at the population level as a result of mutational change in mechanistic parameters: (1) ‘volumes’ of phenotypes in parameter space, (2) ‘distance’ between phenotypes in parameter space, (3) ‘size scale’ of parameter changes between original (donor) and resultant (recipient) phenotypes, and (4) ‘directional bias’ of parameter changes that are more probable in one direction vs. the alternative more entropic direction. The elaboration of these four geometrical factors in the following sections can be visualized in the Design Space that is determined by the architecture of the underlying molecular system (**SI: Sections S5 & S6**).

### Transition Probability Factors

#### Phenotype Volume

Because the volume of a phenotype becomes infinite when the phenotype is independent of some parameter in the model, we bound the universe of values for all parameters by a hyper-cube in log space that is Π-orders of magnitude on edge. The value of Π should be large enough to include all phenotypes in the Design Space but not so large as to exceed physically realistic parameter values; thus, phenotypes that can only be realized with unrealistic parameter values are excluded. We have set Π = 6, which seems large enough to cover all values that can be distinguished experimentally, which is typically about 3-orders of magnitude. For example, the repressor for the lactose operon of *Escherichia coli* binds tightly to specific recognition sites in the DNA with an occupancy of nearly 100%, but reduction of its equilibrium dissociation constant by three orders of magnitude reduces the occupancy to nearly 0% (Lewin 2008). Moreover, it is very unlikely that parameter values ever go to zero because there are typically promiscuous proteins capable of performing the same function with at least some minimal activity (Khersonsky & Tawfin, 2010; Rueda et al. 2019). In any case, we have obtained similar results with Π = 8, and DST3 allows users to select a custom value for Π.

Given a particular set of parameter values characterizing the donor in phenotype volume *V*_*i*_, one of four contributions to the probability of mutating to any other set of parameter values characterizing the recipient in the phenotype volume *V*_*j*_, is given by the ratio of the recipient volume to the total volume for all phenotypes in the repertoire. Thus, this contribution to the probability of mutating from a phenotype with a small volume to one with a large volume is greater than in the opposite direction.

#### Distance Between Phenotypes

We initially consider only mutational events that influence a single parameter at the cellular level. Such mutations can influence multiple systemic functions indirectly, which is pleiotropy at the phenotype level. The mapping from a random change in the digital DNA sequence to the resulting change in the analog value of a kinetic parameter is one of the fundamental mappings that ultimately link genotype to organismal phenotype. Although there is no general understanding of this mapping, the changes in kinetic parameters have a well-defined distance separating their values; regardless of the true distribution of distances produced by the mapping, the PDS framework we are proposing provides a geometrical context for interpreting these distances in relation to the boundaries separating phenotypes, and thus to changes in phenotype. A given *donor* phenotype, which is characterized by a particular set of parameter values, is represented by a unique point within a volume in parameter space (polytope) associated with a qualitatively distinct phenotype. Mutation in a single parameter will result in a new value for the parameter that is represented by a different unique point located in one of a subset of different qualitatively distinct *recipient* phenotypes. The mutation may be forbidden from reaching some qualitatively distinct phenotypes; it also may result in a failure to leave the qualitatively distinct phenotype of the donor (a form of robustness). For example, looking ahead to Fig. 4A, mutations that change the value of the binding constant for the positive transcription factor (*K*_*P*_) of donor phenotype #8 can yield only recipient phenotypes #1, #3, #5, #7 or #8; but not #6, #11, #15 or #16. Mutations that change the value of the binding constant for the negative transcription factor (*K*_*N*_) of donor phenotype #8 can potentially yield any of the recipient phenotypes except for #1 and #5. The distance between phenotypes is rigorously defined by the vertices of the phenotype polytopes and thus is influenced by their shape and orientation (**Fig. S3**).

The single parameter restriction at the cellular level can be relaxed to consider mutations that influence multiple parameters of a single component, which might be considered pleiotropy at the single molecule level (e.g., the degradation rate constant of a transcription factor and its DNA binding constant). The single parameter restriction can also be relaxed to consider simultaneously multiple mutations (e.g., rare double mutant events). However, to make causal predictions upon removal of the single parameter restriction will require formulation of testable mechanistic hypotheses (Lomnitz & Savageau 2016a).

At the population level, the transition between populations of donor and recipient phenotypes typically involves the sum of independent mutational events at the cellular level. For example, this occurs when two different cells, which exhibit the same qualitatively distinct donor phenotype, each undergoes a single mutation, but in different parameters, to yield two different cells that exhibit the same qualitatively distinct recipient phenotype. The transition probability between the two populations is thus the sum of the probability of the two independent mutational events at the cellular level.

#### Size Scale

Large scale mutations are rare; small scale mutations are frequent in well adapted systems (Bataillon & Bailey 2014; Tataru et al. 2017; Bondel et al. 2019; Templeton 2021). This size scale effect depends on the distance, *s*, between the operating point (a parameter set) of the donor phenotype and that of the recipient phenotype. By sampling each donor and recipient combination along the line representing the change in the mutated mechanistic parameter, the probability of each mutation can be calculated based on the volume of the recipient phenotype and the distribution of size scale effects for the mutations. Although, as noted above, the actual distributions for size scale effect are unknown, a reasonable assumption is that the probability of parameter change by mutation decreases exponentially with a size scale λ, i.e., ~ exp(-*s/λ*). This will be made more concrete in RESULTS (*first subsection*).

It may well be that the distribution will be different for different functions, which deep mutational scan experiments might help to clarify. For example, the results for ampicillin resistance in *Escherichia coli* under non-selecting conditions and 100% coverage of change in amino acid residues suggest a nearly normal distribution (Stiffler et al. 2015; Sruthi et al. 2020) with slight asymmetry favoring reduction in activity. Liberles (2023) suggested that these two classes of distributions, exponential and left-truced-at-zero normal, are likely to have similar biological implications, and concluded: “However, it does not actually matter what the distribution looks like as long as the activity level that is being selected is different from the greatest part of the density.”

The size scale effect of mutations can be calculated as an average distance over all combinations of donor and recipient values of the mutated parameter, which is computationally demanding, or by considering the distance between ‘phenotype centroids’, which is analogous to the distance between ‘centers of mass’ for the gravitational force in celestial mechanics. The results are the same for both methods so, since it is computationally more efficient, we use an approximation to the centroid method based on the mid-point of the upper and lower tolerances for each phenotypic volume (red dots in **Fig. S3B,C,D**). The error introduced by this approximation averages less than 10%. Further discussion of this and related issues can be found in the supplemental information (**SI: Section S6, Fig. S5**).

#### Directional Bias

The probability of phenotype-specific transition can be further refined by considering “directional bias”. The probability is larger when a parameter change is in the direction of increasing entropy; it is smaller when the change is in the direction of decreasing entropy. Although the actual differences in value are currently unknown, we account for these directional biases by assigning a multiplicative weighting factor δ that increases the effective size scale λ when a parameter change is in the direction of increasing entropy and decreases it when the parameter change is in the direction of decreasing entropy (**SI: Section S6, Table S1**).

### Phenotype-Specific Mutation Rate Constants

*Phenotype-specific mutation rate constants* refer to transitions at the population level and are determined in three steps. First, for each donor* i* and recipient *j* phenotype, the mechanistic parameter contribution to the mutation, $K_{ij}$, is determined with an exponential distribution (**Table S1**) involving size scale $\lambda$, directional bias $\delta$, and the magnitude of parameter difference *s*, between phenotype centroids $C_{i}$, i.e.,

5 $$ K_{ij} \sim \exp ( - \left| {\log C_{i} - \log C_{j} } \right|/\lambda \delta )\,{\text{or}}\;K_{ij} \sim \exp ( - \left| {\log C_{i} - \log C_{j} } \right|\delta /\lambda ) $$

Depending on whether the mutation involves an increase ($1/\delta$) or decrease ($\delta$) in entropy. The product of the mechanistic contribution, $K_{ij}$, and the volume of the recipient phenotype, *V*_*j*_, is proportional to the probability of a mutation from donor phenotype* i* to recipient phenotype *j*. The $K_{ij}$ are also the sum of independent events when there are multiple paths involving different parameter mutations between the donor* i* and recipient *j* phenotype populations.

Second, the normalized probability of a mutation from donor phenotype *i* to recipient phenotype *j* is written

6 $$ k_{ij} = K_{ij} V_{j} /\left( {\mathop \sum \limits_{j = 1}^{{n_{j} }} K_{ij} Vj} \right)\,{\text{and}}\,\sum\limits_{j = 1}^{{n_{j} }} {k_{ij} } = 1, $$

where *n*_*j*_ is the number of recipient phenotypes that phenotype *i* can reach by independent single mutations in the parameters under consideration.

The *phenotype-specific* mutation rate is proportional to the *general* mutation rate, represented by the parameter *m*. There is a great deal of variation in *m* among species and ecological contexts (Westra et al. 2017). In humans, mutations/base pair is estimated at ~10^–8^ per generation (Nachman & Crowell 2000) and, assuming an average gene size of ~1000 base pairs, this results in a general mutation rate *m* on the order of 10^–5^ mutations/locus per generation. In *E. coli,* mutations/base pair is estimated at ~10^–10^ per generation (Foster, et al. 2015). Thus, for an average gene size, the estimated general mutation rate *m* is on the order of 10^–7^ mutations/locus per generation. Matic et al. (1997) found that values for the *E. coli* mutation rate to drug resistance are typically in agreement with this figure (~10^–7^), but they also found examples as high as ~10^–5^. The values that might have been relevant for early periods of evolution are unknown, but likely to be on the higher end because of error-prone conditions thought to have prevailed at that time. This would be a relevant issue for our case study of a putative primordial circadian clock, which will be treated in RESULTS. We focus on spontaneous point mutations resulting from replication that are the major source of variation in a bacterium like *E. coli* (Foster, et al. 2015). The general mutation rate is subject to evolution in various contexts (Sniegowski et al. 2000; Raynes et al. 2018) and, as we will show for our clock model, different values of the general mutation rate are optimal for individual phenotypes in the context of near-neutral fitness effects (i.e., growth rates, as a measure of total fitness, are nearly equal for all phenotypes).

Finally, the *phenotype*-*specific* mutation rate constant $m_{ij}$ between phenotypes *i* and *j* is given by the product of two factors $mk_{ij}$, where *m* is the *general* mutation rate constant given by the number of mutations per generation and $k_{ij}$ is the probability of a transition from phenotype *i* to phenotype *j*. The production rate of a phenotype (mutant) in units of mutations/time is then the product of $m_{ij}$ the phenotype-specific mutation rate constant, $\gamma_{i}$ the *exponential growth rate constant* of the donor phenotype (related to the doubling time, $\ln 2/\tau_{D}$), and $N_{i}$ the size of the donor population. Note that this differs from the conventional description in that the product $mk_{ij}$ is typically represented by a single *specific* rate constant per generation (e.g., Levin et al. 2000; Reams et al. 2010) that is not predicted but measured or estimated for a particular mutant phenotype.

## Population Dynamic Equations

We initially restrict consideration to asexual haploid organisms in a spatially homogenous context growing in an exponential steady-state, which is the most rigorously defined state for a cellular population (Maaløe & Kjeldgaard 1966). Under these idealized conditions, all effective population sizes *N*_*e*_ are equal to the census population size *N*, mutants are never lost from the population, and the equilibrium distribution can be rigorously determined under non-selecting and selecting conditions. Lethal mutations (~1%) can be subsumed within a net growth rate constant since there is evidence that these mutants occur by a first-order process (Robert et al. 2018).

Of course, steady-state exponential growth cannot continue indefinitely. Nevertheless, results obtained under these conditions provide a rigorous reference or standard to which results under more realistic conditions can be compared, analogous to the historical role played by the frictionless plane in mechanics (Hawking 2002) and by the Hardy–Weinberg law in population genetics (Crow 1988; Wakeley 2005). As with these idealizations, the intention in the present case is to get at something essential with the understanding that refinements will undoubtedly be added in the future; just as wind resistance and static friction were eventually added in mechanics and selection, drift and population structure were eventually added in population genetics. In each case, the expectation is that more realistic aspects will be added as the theory becomes refined. In the DISCUSSION we will suggest methods to relax our initial restrictions.

The population dynamic equations for steady-state exponential growth can be written in terms of numbers *N* for each of the *n* phenotypes in the population:

7 $$ \frac{{dN_{i} }}{dt} = \sum\limits_{\begin{subarray}{l} j = 1 \\ j \ne i \end{subarray} }^{n} {mk_{ji} \gamma_{j} N_{j} } - \sum\limits_{\begin{subarray}{l} j = 1 \\ j \ne i \end{subarray} }^{n} {mk_{ij} \gamma_{i} N_{i} } + \gamma_{i} N_{i} \quad i = 1, \cdots ,n $$

The first sum is the rate of increase by mutation, the second sum the rate of loss by mutation, and the final term the rate of increase by net exponential growth, with $\gamma_{i}$ in doublings per unit time. These equations have the undesirable feature that the population is continually increasing. However, by expressing the population numbers $N_{i}$ as a fraction of the total population $N_{T}$ (or relative frequency) the resulting equations have a more convenient form with a well-defined steady-state. Thus, the relative frequency of phenotype *i* is

8 $$ R_{i} = N_{i} /\mathop \sum \limits_{j = 1}^{n} N_{j} \;{\text{and}}\;\sum\limits_{i = 1}^{n} {R_{i} } = 1 $$

Starting with the derivative of the relative frequency

9 $$ \frac{{d\left( {N_{i} /N_{T} } \right)}}{dt} = \frac{1}{{N_{T} }}\frac{{dN_{i} }}{dt} - \frac{{N_{i} }}{{N_{T} }}\frac{1}{{N_{T} }}\frac{{dN_{T} }}{dt} $$

Substituting $dN_{i} /dt$ from Eq. (7), and noting the cancelation of the mutation terms in $dN_{T} /dt$, we obtain

10 $$ \frac{{dR_{i} }}{dt} = \sum\limits_{\begin{subarray}{l} j = 1 \\ j \ne i \end{subarray} }^{n} {mk_{ji} \gamma_{j} R_{j} } - \sum\limits_{\begin{subarray}{l} j = 1 \\ j \ne i \end{subarray} }^{n} {mk_{ij} \gamma_{i} R_{i} } + \gamma_{i} R_{i} - R_{i} \left( {\sum\limits_{j = 1}^{n} {\gamma_{j} R_{j} } } \right) $$

In anticipation of the case study to follow, we shall consider the situation in which phenotype *k* has growth rate $\gamma_{k}$ in a *non-selecting condition* and $\gamma_{k}^{*}$ in a *selecting condition*. By adding and subtracting the same terms, normalizing time *t* by $\gamma_{k}$($\tau = \gamma_{k} t$ in generations) and defining relative growth rates $\mu_{i} = \gamma_{i} /\gamma_{k}$, Eq. (10) can be rearranged and rewritten for phenotype *k* and for all other phenotypes *i* to emphasize three separate contributions to their rate of change:

11 $$ \frac{{dR_{k} }}{d\tau } = \left[ {\left( {\sum\limits_{\begin{subarray}{l} j = 1 \\ j \ne k \end{subarray} }^{n} {mk_{jk} \mu_{j} R_{j} } } \right) - \left( {\sum\limits_{\begin{subarray}{l} j = 1 \\ j \ne k \end{subarray} }^{n} {mk_{kj} \mu_{k} R_{k} } } \right) + \left( {\mu_{k} - \sum\limits_{j = 1}^{n} {\mu_{j} R_{j} } } \right)R_{k} } \right] - \left[ {(\mu_{k}^{*} - 1)\left( {\sum\limits_{\begin{subarray}{l} j = 1 \\ j \ne k \end{subarray} }^{n} {mk_{kj} R_{k} } } \right)} \right] + \left[ {(\mu_{k}^{*} - 1)(1 - R_{k} )R_{k} } \right] $$

12 $$ \frac{{dR_{i} }}{d\tau } = \left[ {\left( {\mathop \sum \limits_{{\begin{array}{*{20}l} {j = 1} \hfill \\ {j \ne i} \hfill \\ \end{array} }}^{n} mk_{ji} \mu_{j} R_{j} } \right) - \left( {\mathop \sum \limits_{{\begin{array}{*{20}l} {j = 1} \hfill \\ {j \ne i} \hfill \\ \end{array} }}^{n} mk_{ij} \mu_{i} R_{i} } \right) + \left( {\mu_{i} - \mathop \sum \limits_{j = 1}^{n} \mu_{j} R_{j} } \right)R_{i} } \right] + \left[ {(\mu_{k}^{*} - 1)mk_{ki} R_{k} } \right] - \left[ {(\mu_{k}^{*} - 1)R_{k} R_{i} } \right]\,i \ne k, $$

where the seledtion coefficient is defined as $\mu_{k}^{*} - 1$. If there are no fitness effects in the *non-selecting* condition (all growth rates identical), then the form of the above equations in the *selecting* condition has the meaning:

13 $$ {\text{Net Rate of Change }} = {\text{ Mutation}} + {\text{Mutation}} - {\text{x}} - {\text{Selection}} + {\text{Selection}} $$

The middle term involves mutations generated specifically by replication of the phenotype with the selective advantage; hence, it is the only term that involves both a mutation rate and the selection coefficient. The above equations can be considered one of several alternative forms of the standard population genetic equations (Wilke 2005); however, the alternative form used here most clearly reveals the three distinct rate contributions we wish to consider.

## Results

The Design Space Toolbox 3.0 has algorithms for the automatic prediction of numerous characteristics within and between phenotypes. Examples of characteristics *within* phenotypes include the predicted volume (global robustness) of individual phenotypes, protein burden due to differential protein expression, dynamic behavior, and system design principles for the realization of the phenotype. Volumes are shown with identifying phenotype numbers in Fig. 4A. There are numerous phenotypic characteristics that can be plotted on the z-axis as a heat map; an example is the protein burden of each phenotype due to differential protein expression in the non-selecting condition (Fig. 4B). Simulation of the full system, with time* t* scaled by a factor of 1/3 ($\tau = t/3$) to match a 24-h cycle time, produces the results in Fig. 4C, which validates the prediction of a damped oscillatory characteristic for phenotype #7.

Characteristics that distinguish *between* phenotypes include phenotype-specific mutation rate constants and system design principles. In the first case, phenotype-specific mutation rate constants distinguish between phenotypes in the context of dynamics at the population level of organisms with the different phenotypes rather than dynamics of the biochemical molecules of the system (oscillator). In the second case, system design principles distinguish between phenotypes based on the definition of a “qualitatively-distinct phenotype” as a combination of dominant processes operating within an intact biochemical system (Savageau et al 2009). For example, phenotype #5 (signature 11 11 21 11 **11** 11) and phenotype #7 (signature 11 11 21 11 **21** 11) in Table 1 are distinguished by a single change in dominance involving the rate of transcription of the mRNA for the activator (bold digits in the signature). With only the two equilibrium dissociation constants to vary by mutation, the distinction is the following:

14 $$ {\text{Phenotype }}\# {5} \;K_{N} K_{P}^{2} > 316^{2} \; {\text{and}}\;{\text{Phenotype }}\# {7}\;K_{N} K_{P}^{2} < 316^{2} $$

This suggests that a mutation increasing $K_{N}$ alone by a sufficient amount can convert phenotype #7 to #5. In the more general context of distinguishing phenotype #7 from its neighbors, phenotype #7 in Fig. 4A must be to the left of phenotypes #5 and #8 and to the right of phenotypes #3 and #15. The result is not at all obvious or intuitive, rather it is a subtle *system design principle* (Savageau & Fasini, 2009; Savageau 2013) defined by four boundaries (**SI: Section S7**). Thus, all system parameters must satisfy constraints involving specific constellations of values with many opportunities for compensation; there is no single parameter capable of distinguishing between phenotypes. This is particularly apparent in the case of complex diseases for which many genes and parameters interact in subtle ways that are difficult to identify; there is no single effective target for treatment, rather there are many potential targets with a spectrum of effectiveness.

Small changes, in the limit of linearization, within a phenotypic region eliminates the possibility of epistatic interactions. Larger changes, but still within a phenotypic region, can account for a variety of epistatic interactions. For example, the simple conditions in the previous paragraph show an epistatic interaction between two mutations with one affecting $K_{N}$ and the other affecting $K_{P}$. This is clear from the fundamental product of power law nonlinearities found in biochemical kinetics. Moreover, with changes large enough to move the system from one qualitatively distinct phenotypic region to another, nearly any type of epistatic interaction can be realized.

### Fixing The Two Free Parameters λ and δ

Two features that any population model should capture are that “large-effect” mutations are rare whereas “small-effect” mutations are abundant in well adapted systems (Bataillon & Bailey 2014; Tataru et al. 2017; Bondel et al. 2019; Templeton 2021) and detrimental mutations outnumber beneficial ones. Although there are exceptions, which we discuss later, these two features must be considered in the context of a particular model before we can predict phenotype-specific mutation rate constants and fitness effects.

Although terms such as large-, small-, zero-, positive-, and negative-effect are often applied to mutations in describing their effects on *fitness*, these terms only apply to populations in a given environment. With a change in environment the same mutation can have a different, indeed often an opposite, effect on fitness (Templeton 2021). This is because fitness is a property of the phenotype, which in turn is a function of both genotype and environment. To separate these issues, we use the terms “size scale” (i.e., whether the change in value of a kinetic parameter caused by mutation is large or small) and “directional bias” (i.e., whether parameter change caused by mutation is in the direction of increasing or decreasing entropy) to characterize mutations *without regard to fitness*. Fitness is then a function of the environmental context and the phenotype, not of the mutation per se. This separation has the advantage of allowing us to characterize the frequency distribution of phenotypes under non-selecting and selecting conditions.

In the PDS framework, we account for the size scale and directional bias of mutations with an exponential distribution having scale factor λ and directional bias parameter δ that increases or decreases the effective scale factor. Unlike the other parameters in this theoretical framework, these two must be estimated from experimental data. For this purpose, we draw upon the best studied specific function in molecular biology, LAC repressor binding to its recognition sites in the DNA of *E. coli.* Markiewicz et al. (1994) generated ~ 4000 protein variants by making substitutions at each amino acid position. After being transformed through the molecular mechanisms that provide the causal connection between the gene sequence and the integrated function of the *lac* system, a corresponding distribution of phenotypes was determined. As Markiewicz et al. (1994) showed, there are essentially three qualitatively distinct phenotypes involving LAC binding: (1) the inducible “wild-type”, (2) non-inducible constitutive, and (3) non-inducible super-repressed. Under the conventional laboratory conditions used to detect these three phenotypes, the data in their Fig. 1 show that changes at ~67% of the positions were tolerant to substitutions (no change in DNA binding), 31% were intolerant with an increase in binding entropy (decrease in DNA binding), and ~2% were intolerant with a decrease in entropy (increase in DNA binding).

Markiewicz et al. (1994) suggested that this distribution is likely to be similar for other proteins. For example, they examined the sequence alignment of proteins in the LAC family of proteins (which includes proteins of unrelated function in addition to other transcription factors) and found that 61% of the residues were not conserved (tolerant of evolutionary changes) and 39% were conserved (intolerant of evolutionary changes).

The actual distribution will undoubtedly be different for different functions, organisms and contexts, which deep mutational scan experiments might help to clarify. For example, the results for ampicillin resistance suggests that the ratio of negative to positive effects in the non-selecting condition is approximately twofold (Stiffler et al. 2015: Fig. 3A), which is smaller than the 15-fold value observed for the LAC repressor. The value of δ, which is fitted over all relevant qualitatively distinct phenotypes will also be less. Similarly, the size scale parameter for the quantitative distribution in the ampicillin case (Log10 σ = 0.07 of a log unit) is smaller than that estimated for the LAC case (λ = 0.6 of a log unit). However, the percent of conserved residues in the ampicillin case is 35% (Sruthi et al. 2020: Table 1) is similar to that in the LAC case (33% = 31% + 2%), which suggests that the ampicillin data are no consistent: the percentage of conserved residues (similar to LAC) but the size scale very different (much smaller than LAC). Sruthi et al. (2020) analyzed conservation for six proteins with partial coverage of residue changes from *E. coli* (1), *Streptomyces sp* (1), *Saccharomyces cerevisiae* (1) and *Homo sapiens* (3) and found a mean of 60% with a standard deviation of 20%, which is consistent with the notion that distributions will be different for different functions, organisms and contexts. The implications for the clock repressor are unclear, particularly at the unknown presumptive early stage in its evolution assumed here as compared to existing highly evolved clocks.

In the prediction of phenotypes resulting from mutations in the N gene of the clock model, there are three qualitatively distinct phenotypes analogous to the LAC case: the oscillatory “wild type”, the non-oscillatory constitutive, and non-oscillatory super-repressed (**Fig. S6**). Although the distribution among these would be unknown, let us assume for our case study that these have the same distribution as the LAC repressor. Values of λ = 0.6 and δ = 1.85 then provide the best fit to the experimental data and the predicted distribution of fitness effects in this case is ~ 67% oscillatory (wild-type DNA binding), ~ 31% non-oscillatory constitutive (decreased DNA binding), and ~ 2% non-oscillatory super-repressed (increased DNA binding).

To summarize, there are two free parameters in this model, λ and δ, that must be estimated from experimental data. Based on the above considerations, for our case study we assign the following model values for these two parameters: λ = 0.6 and δ = 1.85. All the remaining parameters have values predicted solely based on the underlying mechanistic model using methods from the Design Space Toolbox (Valderrama-Gómez et al 2020) and used for further predictions, as described in the following sections.

### Predicting The Equilibrium Distribution of Phenotype Diversity

In what follows we predict the equilibrium distribution of phenotype diversity under non-selecting conditions in three stages to clearly distinguish different contributions. First, we consider the idealized case in which there is no size scale or directional bias for mutations that have neutral fitness effects and show that the distribution differs from the expectation of a uniform distribution. Second, we add size scale and directional bias and find that the distribution exhibits an increasing gradient from phenotypes with low entropy to those with high entropy. Third, as a specific example involving phenotypes with mixed fitness effects, we consider their protein burdens to obtain a distribution with a central peak resulting from *entropy – selection balance*. It should be noted that this type of balance is different from other types of specific mutation – selection balance (Barton 2007; Lynch 2010; Orlenko et al. 2016b) and the general mutation – selection balance that always exists at equilibrium. Finally, we illustrate the shift in the distribution when the oscillatory phenotype is subject to various degrees of selection.

#### Distributions for Neutral Mutations Without Size Scale or Directional Bias Effects

**Fig. 5:**
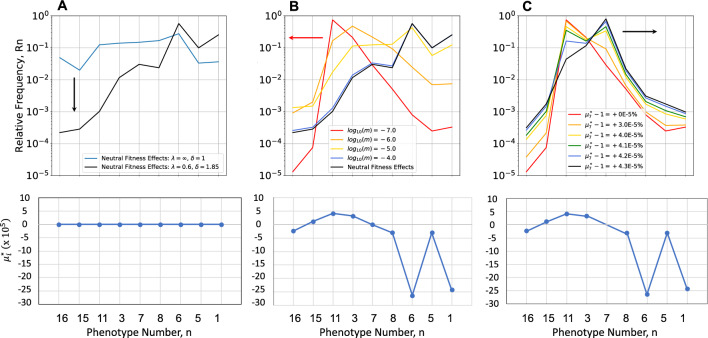
Equilibrium distributions of phenotype diversity. Mutational entropy is increasing from left to right, from the phenotype with both equilibrium dissociation constants having the lowest values (phenotype #16) to that with both having the highest values (phenotype #1). Fitness effects are shown in the lower panels. **A** Mutations with neutral fitness effects (all $\mu_{i} = 1$) under non-selecting conditions (Blue) in the absence of size scale ($\lambda \to \infty$) and directional bias ($\delta = 1$), and shifted down (Black) in the presence of size scale ($\lambda = 0.6$) and directional bias ($\delta = 1.85$). In the absence of directional bias there is a minimal gradient; whereas this gradient is approximately 4-orders of magnitude when directional bias is present. **B** Mutations with mixed fitness effects ($\mu_{i}$ different) under non-selecting conditions in the presence of size scale ($\lambda = 0.6$) and directional bias ($\delta = 1.85$). The distribution is shifted to the left with decreasing values of *m* = 10^–4^ (Blue), 10^–5^ (Yellow), 10^–6^ (Orange) and 10^–7^ (Red) compared with the strictly neutral results in (Black). The distribution changes dramatically, increasing, reaching a peak, and then decreasing when directional bias is present. Fitness effects normalized with respect to the experimental data for *E. coli* ß-galactosidase burden. **C** Mutations with mixed fitness effects ($\mu_{i}$ different) under selecting conditions with various degrees of selection. The peak of the distribution under the non-selecting conditions (*m* = 10^–7^) shifts to the right, from phenotype #11 (non-oscillatory, Red) to phenotype #7 (oscillatory, Black) and its frequency increases with increasing values of the selection coefficient whereas the frequency of the other phenotypes decrease according to their selective disadvantage

Neutral mutations without size scale or directional bias effects produce a uniform distribution of values in parameter space; however, the partitioning of Design Space, which is dictated by the architecture of the underlying molecular system, results in an equilibrium distribution of phenotype frequencies that is determined by the normalized values of the phenotypic volumes (Fig. 5A**,** Blue), as obtained analytically. Large volumes (e.g., phenotype #6) imply robust phenotypes that are tolerant to large changes in parameter values; small volumes (e.g., phenotype #15) imply fragile phenotypes that are easily disrupted by small changes. The absence of size scale and directional bias is of course an idealization, but useful for identifying the volume contribution and providing a baseline on which to characterize more realistic features, as described below.

#### Distributions for Neutral Mutations with Size Scale and Directional Bias Effects

In the presence of size scale and directional bias effects (λ = 0.6 and δ = 1.85), the equilibrium distribution exhibits a gradient from phenotypes with lower entropy (lower left corner in Fig. 4A) toward phenotypes with higher entropy (upper right corner in Fig. 4A), as obtained numerically from the steady-state solution of the population dynamic equations and shown in Fig. 5A** (**Black**)**. Note that the phenotype with highest entropy, based on directional bias, is phenotype #1, which corresponds to mutations in both transcription factors that essentially eliminate the ability to recognize their DNA binding sites. Conversely, the phenotype with the lowest entropy, based on directional bias, is phenotype #16, which corresponds to mutations in both transcription factors that make for overly tight binding. The gradient in this case is approximately 4-orders of magnitude.

#### Distributions for Mixed Mutations with Size Scale and Directional Bias Effects

In the non-selecting constant light environment, in which mutants are assumed to exhibit fitness differences unrelated to the specific phenotype characteristic of oscillation, the equilibrium distribution is among mutations with mixed fitness effects, positive, negative and neutral. As an example of a phenotype- specific fitness characteristic that can be predicted, we consider the size of the protein coding regions and the protein burden of extraneous protein expression for each phenotype.

Experimental evidence in the case of *lac* operon expression in *E. coli* suggests that inappropriate constitutive expression (nevertheless within the normal range for expression of the wild-type-induced state) decreases the growth rate by <  ~ 0.1%. (Koch 1983). The decrease would be even less if we consider only the contribution from ß-galactosidase, and neglecting that from the permease and transacetylase, in making estimates for our clock module. Given the tenfold larger size of the ß-galactosidase monomer, its tetrameric structure and the 1000-fold protein burden (difference between wild-type uninduced expressed and mutant constitutive expression), compared to the assumed 100 amino acid length, dimer structure and predicted 100-fold protein burden for our molecular model, allows the appropriately scaled decrease in growth rate to be <  ~ 0.001%. The following relative growth rates (fitness effects) for each phenotype, relative to phenotype #7 in the non-selecting condition, follow from the predicted levels of protein expression for each phenotype (Fig. 5B): $\mu_{1}$ = 0.999997573 (-2.43E-04%), $\mu_{3}$ = 1.000000322 (3.22E-05%), $\mu_{5}$ = 0.999997527 (-2.47E-04%), $\mu_{6}$ = 0.999997357 (-2.64E-04%), $\mu_{7}$ = 1.0 (0%), $\mu_{8}$ = 0.999999693 (-3.07E-05%), $\mu_{11}$ = 1.000000412 (4.12E-05%), $\mu_{15}$ = 1.000000115 (1.15E-05%), and $\mu_{16}$ = 0.99999976 ( -2.40E-05%). Note that these small differences in growth rate that are undoubtedly overestimates would be considered neutral, given the technical limitations of experimentally determining growth rate differences less than ~ 0.1% (Gallet et al. 2012).

When both size scale and directional bias effects are present, the graded distribution in the strictly neutral case (Fig. 5A,B**,** Black) is dramatically changed to a peaked distribution that is increasingly weighted to the left (Fig. 5B Orange, Red) as the general mutation rate is decreased. The result is what might be called *entropy-selection balance*.

Note that all the distributions in Fig. 5A and 5B occur under the *non-selecting condition with respect to the oscillatory* phenotype characteristic. Moreover, despite the difficulty distinguishing between mutations with neutral fitness effects and mutations without detectable fitness effects, these results show that the equilibrium distributions are radically different. It is also clear that there is an optimal value for the general mutation rate that favors each phenotype.

#### Equilibrium Distribution of Phenotype Diversity Under The Selecting Condition

When connections to both the synchronizing environmental signal and the integrated cellular biochemistry are made by a critical new mutation, it would confer no selective advantage if it were to occur in one of the phenotypic regions that lack the ability to oscillate. For example, it has the highest probability of occurring in phenotype #11 because its frequency in the population is nearly 100% before the mutation occurred. More rarely, it would occur in the region of phenotype #7, but then there would be the potential to synchronize with the light–dark environment (the selecting condition) and have a selective advantage. The predicted equilibrium distribution of phenotype diversity under the selecting condition as a function of the selection strength is shown in Fig. 5C. Beyond a critical level of selection, the peak of the equilibrium distribution shifts from phenotype #11 to phenotype #7. Although we cannot currently predict the fitness of phenotype #7 under selecting conditions, if it were possible to estimate the distribution of phenotype diversity (e.g., from deep mutational scan experiments), then one could back calculate the selection strength that produces the best fit to the estimated distribution (Fig. 9 and **SI: Section S8, Fig. S7**).

**Fig. 6:**
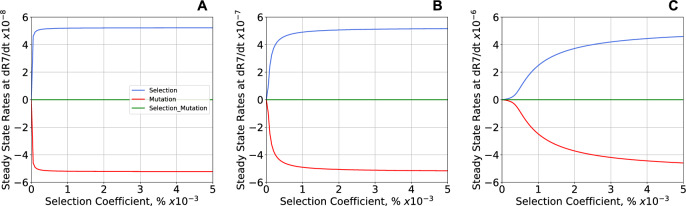
Three separate contributions to the steady-state rate of change in frequency for the oscillatory phenotype #7. The three panels show results for mutations with neutral fitness effects and general mutation rate **A** *m* = 10^–7^, **B** *m* = 10^–6^, and **C** *m* = 10^–5^. The contributions (**Eq. **13) are shown as a function of selection strength at equilibrium. Selection alone (Blue) is balanced with mutation alone (Red); the contribution by mutation-x-selection (Green) is negligible for the strengths of selection shown. The maximum rates of change are proportional to the general mutation rate (note the change of scales), and stronger selection is required to overcome the effects of higher general mutation rates

The three separate contributions to the rate of change in phenotype frequency in the neutral case (**Eq. **13, mutation, mutation-x-selection, and selection) are shown in Fig. 6 as a function of selection strength and general mutation rate. The rate of change at equilibrium is equal to zero and the contributions of mutation alone and selection alone are nearly opposite and equal. The contribution from mutation-x-selection is negligible at the selection strengths shown. Note the differences in scale: the maximum contribution to the rate at equilibrium is proportional to the general mutation rate, and the degree of selection necessary to achieve the maximum rate increases rapidly with the general mutation rate.

### Non-Equilibrium Distribution of Phenotypes Under the Selecting Condition

In this and the following subsection, instead of determining the phenotype distribution at equilibrium under either the non-selecting or selecting condition, we determine the temporal changes in distribution during the transition between the two equilibria – from non-selecting to selecting or from selecting to non- selecting. The light–dark environment generates the selecting condition. The ability to synchronize with the light–dark environment generates a selective advantage for the oscillatory phenotype (#7) greater than that of the other phenotypes. Aside from #7, all the other phenotypes have either a mixed distribution or a neutral distribution of fitness effects.

**Fig. 7:**
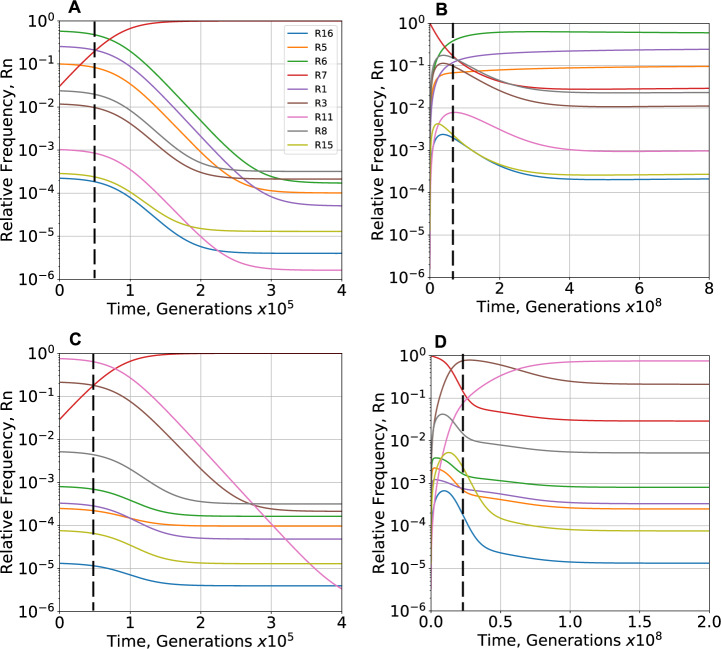
Temporal response in relative frequency of phenotypes following imposition and removal of the selecting condition. **A**, **B** Neutral distribution of fitness effects. **A** The increase of phenotype #7 (Red) is accompanied initially by very little change in the other phenotypes, followed (after the dashed line) by a slow decrease in all other phenotypes. All changes in the rank of the relative frequencies occur before 3.5E + 05 generations. **B** The decrease of phenotype #7 (Red) is accompanied initially by a rapid increase in all other phenotypes, a peak (the last occurring at the dashed line) followed by a slow decrease in all other phenotypes except for phenotypes #1, #5 and #6, which continue to increase slowly. All changes in the rank of the relative frequencies occur before 2.5E + 08 generations. The overall response is ~ 1000-times slower than **A**. **C, D** Mixed distribution of fitness effects. **C** The increase of phenotype #7 (Red) is accompanied initially by very little change in the other phenotypes, followed (after the dashed line) by a slow decrease in all other phenotypes. All changes in the rank of the relative frequencies occur before 4.0E + 05 generations. **D** The decrease of phenotype #7 (Red) is accompanied initially by a rapid increase in all other phenotypes, a peak (the last occurring at the dashed line) followed by a slow decrease in all other phenotypes except for phenotype #11, which continues to increase. All changes in the rank of the relative frequencies occur before 6.3E + 07 generations. The overall response is ~ 400-times slower than **C**. Imposition occurs by a change from a non-selecting (μ_7_* = 1.0) to a selecting (μ_7_* = 1.00006) environment and removal by the reverse. The general mutation rate *m* = 10^–7^

Results with a neutral distribution of fitness effects for phenotypes other than #7 are shown in Fig. 7A, starting from the equilibrium distribution under the non-selecting condition (Fig. 5A,B: Black) and evolving to the equilibrium distribution under the selecting condition (selection coefficient μ_7_^*^—1 = 6.0E-3%, all other μ_*i*_ = μ_7_ and fixed). Phenotype #7 increases rapidly with a time scale dominated by selection, while there is little change in the other phenotypes until ~ 5.0E + 04 generations (Fig. 7A**,** vertical dashed line). After this point, phenotype #7 approaches its maximum at ~ 1.5E + 05 and all other phenotypes slowly decrease asymptotically toward the new equilibrium distribution with a time scale dominated by mutation. There are no changes in the ranking of phenotype frequencies in the population after 3.5E + 05 generations.

### Non-Equilibrium Distribution of Phenotypes with Removal of the Selecting Condition

Experimental studies have explored the evolutionary loss of phenotypes in response to the relaxation of selection. For example, the ability of *Bacillus subtilis* to sporulate is lost when it is no longer under selection (Maughan et al. 2007). In the clock model, relaxation of selection occurs when the selective advantage of phenotype #7 is removed (switched to constant light) and the population returns with time to the equilibrium distribution under the non-selecting condition.

Results with a neutral distribution of fitness effects for phenotypes other than #7 are shown in Fig. 7B, starting from the equilibrium distribution under the selecting condition (selection coefficient μ_7_^*^—1 = 6.0E-3%, all other μ_*i*_ = μ_7_ and fixed) and evolving to the distribution under the non-selecting condition (Fig. 5A,B: Black). The large number of the selected phenotype (#7) in the initial equilibrium distribution is rapidly lost and redistributed to all the other phenotypes within ~ 7.5E + 07 generations. There is a subsequent slow redistribution and decrease among all the phenotypes except #1, #5 and #6 (high entropy phenotypes) until a new equilibrium distribution is approached asymptotically with a time scale dominated by mutation. There are no changes in the ranking of phenotype frequencies in the population after ~ 2.5E + 08 generations. Comparison of the time scales in Fig. 7A and B shows that the response to the removal of selection is approximately ~ 1000-times slower than that to the imposition of selection.

Results with a mixed distribution of fitness effects for phenotypes other than #7 are shown in Fig. 7D, starting from the equilibrium distribution under the selecting condition (selection coefficient μ_7_^*^—1 = 6.0E-3%, all other μ_*i*_ determined by protein burden and fixed) and evolving to the distribution under the non-selecting condition (Fig. 5B: red). The large number of the selected phenotype (#7) in the initial equilibrium distribution is rapidly lost and redistributed to all the other phenotypes within ~2.5E + 07 generations. There is a subsequent slow redistribution and decrease among all the phenotypes except #11 (low entropy phenotype) until a new equilibrium distribution is approached asymptotically with a time scale dominated by mutation. There are no changes in the ranking of phenotype frequencies in the population after ~6.3E + 07 generations. These results are in qualitative agreement with those of Maughan et al. (2007) when the larger target size of the sporulation machinery and the higher mutation rate of their mutator strain are considered. Comparison of the time scales in Fig. 7C and D shows that the response to the removal of selection is approximately ~400-times slower than that to the imposition of selection.

The large differences in time scale indicate that alternating between equal periods in selecting and non-selecting environments before reaching equilibria would lead not to an average of the two distributions but to a distribution closer to that in the selecting environment, which is reminiscent of “conflict between selection in two directions” (Haldane & Jayakar 1963).

## Experimental Implications

There are two major challenges in determining the distribution of phenotypes available for selection to act upon. One is the time of sampling relative to the evolutionary dynamics of natural populations and the second is technical limitations in the ability to identify and measure phenotypes. Both help to explain the pessimism expressed by Charlesworth (1996) in determining the distribution of phenotypes and their fitness characteristics in natural populations.

Experimental studies based on mutants constructed from a highly evolved system (wild type) in a given environment (in the extreme, optimized according to Fisher’s Geometric model) may have only a very narrow distribution of alternative phenotypes capable of improvement in that environment. Those based on mutants constructed from a system that is far from its optimal state in a new environment, are likely to offer a more fertile distribution of phenotypes capable of improvement. Indeed, Matuszewski et al. (2014) pointed out a violation of Fisher’s prediction that mutations of small effect are the primary raw material of adaptive evolution. They considered a geometric model like Fisher’s but with environmental change. In contrast to Fisher’s predictions, larger adaptive steps often occur with a moving optimum. Mutations of small effect are not always the main material of adaptive change even when there is a single adaptive optimum, albeit a moving one. However, determining the natural distribution from subsequent measurements depends on the time of sampling following the construction, with the actual distribution of fitness effects bounded by two extremes: sampling at time zero and sampling at the time to reach equilibrium. The time zero sample has not involved any exchange; thus, it simply reflects the construction and may have little to do with any subsequent distribution in nature. The equilibrium sample in some cases might be the more relevant distribution in nature, but there is insufficient time to test this in practice. Thus, the natural distribution undoubtedly lies somewhere between these extremes. There is the additional difficulty of identifying the phenotypes because of technical limitations. Experimental studies based on natural variants face the same two challenges.

Orr (2005) also identifies challenges in two related problems. “The first is the current theory is limited in several ways – all the models that have been mentioned rest on important assumptions and idealizations. Although they are reasonable starting points for theory, none of these assumptions is necessarily correct and changing any might well change our predictions. […] The second problem concerns testability. The difficulty is practical, not principled. Whereas current theory does make testable predictions, the effort required to perform these tests is often enormous (particularly as the theory is probabilistic, making predictions over many realizations of adaptation). Given, for example, the inevitable and often severe limits on replication in microbial evolution work, we can usually do no more than test qualitative predictions.” Our theory is grounded in measurable biochemical parameters, and thus a different set of assumptions and idealizations need experimental testing.

### Experimental Evolution Studies in a Chemostat

**Fig. 8:**
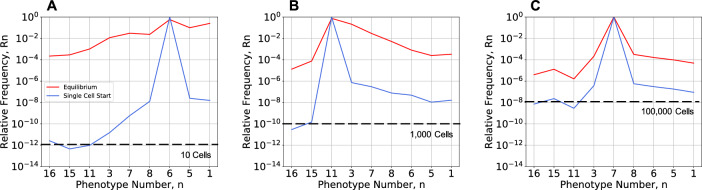
Non-equilibrium distributions of phenotype diversity under non-selecting and selecting conditions after exponential growth from one to 10^13^ cells. Cells with general mutation rate *m* = 10^–7^ are inoculated into fresh media in a one-liter chemostat without flow. **A** Under non-selecting conditions with neutral fitness effects, phenotypes with the lowest frequency (#11, #15 and #16) are expected to have ~10 cells in the chemostat. **B** Under non-selecting conditions with a protein burden spectrum of fitness effects, phenotypes with the lowest frequency (#15 and #16) are expected to number ~1000 cells. **C** Under selecting conditions with a protein burden spectrum of fitness effects, nearly all phenotypes are expected to be present at more than ~100,000 cells. Size scale effects and directional bias effects are present in all cases. The initial distributions (Blue) can be expected to approach the equilibrium distributions (Red) asymptotically with time following long-term exponential growth with the flow of fresh media to the chemostat

The equilibrium distributions of phenotype diversity under selecting and non-selecting conditions can be approximated experimentally by growing populations in a chemostat/turbidostat (Bustos & Golden 1992; Gresham & Jong 2015). This allows us to relax the assumptions concerning the ideal context. If a one- liter chemostat is initialized with a single cell and the population grows exponentially until reaching typical densities of 10^8^ to 10^10^ cells/ml (Gresham & Jong 2015), at this point nearly all phenotypes will be present in the population (Fig. 8). If the flow of fresh media into the chemostat is initiated at this point, the doubling of the population in each subsequent generation due to growth coupled with the 50% reduction in population size per generation due to dilution will introduce fluctuations in the numbers of cells. Phenotypes with a low frequency must be treated stochastically when the differences between effective population size and the census population size become significant. All other phenotypes are expected to persist in the chemostat.

Under non-selecting conditions, the case with neutral fitness effects is the most difficult. At the time when the chemostat reaches the full operating density, all phenotypes in the population will be present with a significant frequency except for #11, #15 and #16 (~ 10 cells in Fig. 8A). The issue of genetic drift could be introduced here by the addition of stochastic changes (replication vs. removal) in each generation. In the case of mixed fitness effects due to protein burden, even those phenotypes with the smallest frequency will have a census size of ~ 1000 cells (Fig. 8B). Under selecting conditions, at the time when the chemostat reaches the full operating density, even those phenotypes with the smallest frequency will have a census size of ~ 100,000 cells (Fig. 8C).

### Measuring Qualitatively Distinct Phenotypes

Although current experimental limitations make it difficult to measure individual phenotypes, there are some cases in which relevant aggregate phenotypes can be measured. In the classic studies of Markiewicz et al. (1994), the authors constructed a collection of LAC mutants, measured their β-galactosidase expression, grouped the results into qualitatively distinct phenotypes (constitutive, super-repressed or inducible), and determined the resulting distribution of phenotypes *measured at time zero*. They found 2% super-repressed, 67% inducible, and 31% constitutive. This is not surprising, given a low mutation rate (*m* = 10^–7^) and that the construction started with the highly evolved and presumably fit *lac* system of *E. coli*.

**Fig. 9:**
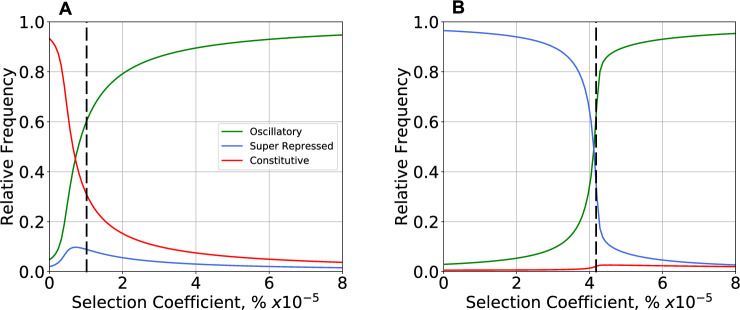
Equilibrium distributions of qualitatively distinct phenotypes under selecting conditions with various degrees of selection. Comparisons made with general mutation rate *m* = 10^–7^ and **A** neutral fitness effects **(**all $\mu_{i} = 1$), and **B** a protein burden spectrum of fitness effects ($\mu_{i}$ different) under non-selecting conditions ($\mu_{7}^{*} = 1$). The degree of selection required to reach a distribution with 60% oscillatory phenotype (dashed line) with mixed fitness effects is ~ four-fold greater than that with neutral fitness effects. The second most common phenotype is super-repressed with mixed and constitutive with neutral fitness effects. Thus, only the results predicted in **A** match the experimental results of Markiewicz et al. (1994)

Fluorescently tagged protein might be an updated approach for other proteins. In our case study, measuring the activity of the N gene protein and classifying the results as constitutive (phenotypes #6 and #8), super-repressed (phenotypes #3 and #11) or oscillatory (phenotype #7) leads to the following predictions. In analogy with the LAC studies, and sampling the distribution at time zero, our results would match those of Markiewicz et al. (1994) because these values were used to fit the two free parameters of our model λ and δ. The distribution of phenotypes measured after reaching equilibrium under non-selecting conditions (loss of selection) with *neutral fitness effects* is predicted to be 2% super-repressed, 5% oscillatory and 93% constitutive (Fig. 9A**,** $\mu_{7}^{*} = 1$). This reflects the dominant influence of entropy. The distribution of phenotypes measured after reaching equilibrium under non-selecting conditions with *mixed fitness effects* based on protein burden is predicted to be 96% super-repressed, 3% oscillatory and 1% constitutive (Fig. 9B**,** $\mu_{7}^{*} = 1$). Under selecting conditions, the degree of selection required to reach a distribution with 60% oscillatory phenotype with mixed fitness effects is four-fold greater than that with neutral fitness effects. These differences, suggesting that the results with a neutral distribution of fitness effects can be achieved more easily than with the protein burden distribution, might be relevant for the evolution of LAC repressor as well. Furthermore, an examination of different values for the general mutation rate, *m*, at equilibrium with mixed fitness effects shows that even when the relative frequency of the oscillatory phenotype is maximum at *m* = 3 × 10^–6^, the results are still very different from that of wild-type LAC repressor selected in nature (**SI: Section S8, Fig. S7).**

Testing such predictions would require finding rare cells in the population, at the limit of detection for many methods. Based on the start of a chemostat experiment as described in the previous section, the effluent at any subsequent time during the experimental evolution could be collected and the cells subjected to counting or sorting. Counting might well be able to determine the numbers of rare cells, sorting would allow sufficient material for further experimental tests. A double-sieve strategy would have advantages. First, the cells are grown under *non-selecting* conditions and sorted into two abundant classes, those with constitutive and non-constitutive expression. Second, the sorted cells with non-constitutive expression are grown under *selecting* conditions and sorted into those enriched for super-repressed and wild-type expression. This approach would require ~ 10^10^ cells to be collected and sorted within a reasonable amount of time and cost, which should be feasible with recent advances in high-throughput sorting methods (Fan et al. 2013; Zhukov et al. 2021).

## Discussion

Two complex and interrelated issues in evolution are the *distribution of phenotype diversity*, which offers opportunities for innovation, and the *interaction of phenotype-specific mutation rates and phenotype fitness differences*, which determines population dynamics and the subsequent evolution of the population. Some experimental approaches to determining the distribution of mutant effects only address large effect mutations because there are technical limitations to the size of changes in growth rate that can be measured (Gallet et al. 2012). Others only address small effect mutations in the context of nearly neutral theory (Kimura 1983; Ohta 1992). As Bondel, et al. (2019) pointed out, together the two provide a bigger picture by complementing one another. However, neither of these approaches deal with the causal linkages between genotype/environment and phenotype.

There are few examples attempting to determine the distribution of mutant effects by addressing the mechanistic link, and they use a simulation-centric approach that differs methodologically from the phenotype-centric approach used in the PDS framework. Orlenko et al. (2016a; 2016b) have examined unbranched pathways in which classical Michaelis–Menten kinetics were assumed, kinetic parameters were sampled, and the system of ordinary differential equations was repeatedly solved. They note that more complex realistic systems remain to be studied in this context. Examples include systems involving more complex forms of regulation, enzyme-enzyme complexes and cascades, as well as branched and cyclic pathways. Loewe & Hillston (2008) focused on the simple limit cycle model of Leloup, et al. (1999) for circadian rhythms with a set of assumed parameter values as reference. They converted the biochemical kinetic equations from ordinary differential equations into pseudo-chemical kinetic equations for stochastic simulations. They employed dense sampling of parameter values and repeated stochastic simulations to generate statistical data for analysis in terms of various fitness correlates. Brajesh et al. (2019) focused on the *lac* operon of *E. coli* because it is a simple, specific system that has been studied for decades (Muller-Hill 1996; Ullmann 2003) and for which there are experimental values for nearly all the key parameters. Starting with this well-characterized system, they explored its phenotypic repertoire by dense sampling of the parameter space combined with numerical solution of the ordinary differential equations for the nonlinear mechanistic model. It will be difficult to replicate these approaches for other systems in which there are a large number of parameters with unknown value that are difficult or currently impossible to measure or estimate. This is precisely the bottleneck currently limiting the successful application of the conventional simulation-centric modeling strategy. This ultimately becomes a scaling issue for large systems because of the magnitude of sampling required, coupled with the repeated deterministic and stochastic numerical simulations of the nonlinear differential equations. Moreover, with certain combinations of parameter values these numerical solutions often fail for technical reasons (e.g., ‘stiffness’ of the nonlinear kinetic equations), which makes automation of the process problematic.

The phenotype-centric modeling strategy largely circumvents the bottleneck presented by a mechanistic model with a large number of unknown parameter values (Valderrama-Gómez & Savageau, 2018). Here we showed that it also can predict phenotype-specific mutation rates and the distribution of mutant effects under non-selecting and selecting conditions. It must be noted that the phenotype-centric approach does not escape the issue of scaling to large realistic systems, although it does not involve the limitations of sampling and repeated simulation mentioned above. The issue is the large number of phenotypes that must be treated analytically for any realistic system. However, each phenotype is a separate linear algebraic problem, which makes it what computer scientists call ‘embarrassingly parallelizable’, and therefore amenable to cloud computing.

By way of conclusion, we discuss differences between the PSD theoretical framework and other theoretical frameworks, similarities between them, and potential areas of mutual interest for further development. We finish with a summary of results, some that are consistent with well-known results in theoretical population genetics and others that are new.

### Differences and Similarities Between Theoretical Frameworks

The broad context of theoretical population genetics is found in the historical review of Orr (2005). He focused on the advances and limitations involving the two main classes of mathematical models: older phenotype-based models following in the spirit of Fisher’s Geometric model and newer DNA sequence-based models emphasizing nearly neutral and extreme value theory.

The Phenotype Design Space model has some superficial similarity to the Geometric model of Fisher (1930), but it is fundamentally different. Although both prominently feature *geometry, quantitative phenotype traits* and *size of mutational changes*, a brief comparison of Fisher’s Geometric model vs. the PDS model shows there is little else in common:

- Phenotype definition is *generic*, *descriptive, and ad hoc* (height, weight, etc.) vs. *specific*, *mathematical, and rigorous* (genetically determined parameters and environmentally determined variables).
- Phenotypic traits are for *unspecified systems* in *unstructured Cartesian space* vs. *biochemically specified systems in structured logarithmic space*.
- Mutation causing *symmetric* changes involving *any combination of the orthogonal traits (omnidirectional)* vs. *asymmetric* (entropic) changes involving *one mechanistic trait (bidirectional)*.
- Mutations simultaneously affect *all n traitsl* (*general* pleiotropic) vs. a single *specific trait* (model-dependent pleiotropic).
- Organizing principle is *random variation in proximity to an optimum* vs. *deterministic structure of a global Design Space*.
- Methodology focused on *statistical analysis and computer simulation* vs. *analytic geometry and computational algebra*.
- Focus on new mutations vs. standing genetic variation.

Although these theories are very different, there are a few connections between them that might be worth exploring. For example, two strong results from Fisher’s model and the extreme value theory are that an exponential distribution of positive effect mutations may be universal (Orr 2005) and that there is a progression of size effects from initially large to subsequently smaller (Gillespie 2004). In PDS theory, the first of these results might have a connection to the assumption of exponential distributions for both positive and negative-effect mutations. However, these exponential distributions are in logarithmic coordinates, which in Design Space theory means that they could also be considered power law in Cartesian coordinates. Regarding the second of the above results, we can speculate that if the initial mutation takes the system from an optimal state into a qualitatively different region of Design Space, then the first significant mutation taking it back will likely have a large effect on average. Once back near the optimum, then smaller quantitative changes will add refinements. However, back mutations with small changes at the level of kinetic parameters could lead to large qualitative changes at the phenotype level, but only when the phenotype undergoing back mutation is quantitatively near the common boundary with the recipient phenotype. This is also related to the long-standing robustness vs. evolvability issue (de Visser et al. 2003; Draghi et al. 2010; Payne & Wagner 2014; Greenbury et al. 2016; Wei & Zhang 2017). In our mechanistic framework phenotypes with large volumes in Design Space are globally the most robust to mutation (to changes in the qualitatively distinct phenotype). Mutations with large-size effects can explore distant phenotypes infrequently. However, if there is a more favorable adjacent phenotype, then there will always be a minority of cells with parameters that locate them near the boundary with the more favorable phenotype so that even mutations with small-size effects can result in movement into a qualitatively different phenotypic region that is favorable. Thus, evolvability coexists with robustness. A statistical approach within the PDS framework could be used to test these speculations.

The results in Fig. 8, which represent the most extreme bottleneck with a single founder cell, suggest that most of the phenotypes are regenerated with sizable cell numbers within the initial growth phase. A stochastic approach could be used to study the long-term fate of the remaining phenotypes whose population sizes are <  ~100 cells, each of which is retained or lost in each generation.

Liberles (2023) reviewed problems inherent in the common assumption that mutational effects will be symmetrically distributed about a static mean (as in Fisher’s model) and, conversely, calls attention to the under-appreciated ideas of Constructive Neutral Evolution (Stoltzfus 1999; Muñoz-Gómez et al. 2021) that has its roots in biased (asymmetrical) mutational processes.

The key concepts of CNE (Stoltzfus 1999) tend to be general, descriptive and qualitative but have some similarity to specific quantitative aspects of our PDS framework, as shown in the following comparisons.

- Biased variation (*via* mutational machinery) *in CNE is made concrete and quantitative in the PDS treatment of bias*
- Biased variation (*via* systemic aspects of organization and interaction) *in CNE is made concrete and quantitative in the PDS treatment of relative volumes and global robustness of phenotypes*
- Excess capacity *in CNE is made concrete at the precursor stage in any PDS analysis, as discussed in the introduction of the model in *Fig. 3
- Epistasis *in CNE occurs when effects of a mutation are dependent on the context provided by other mutations or genes and is a consequence of excess capacities, e.g. the result of gene duplication, whereas in the PDS framework epistasis is quantitative and specific to the integrated system in question*

The contrast between the neutral (symmetrical) and biased (asymmetrical) views is especially apparent in the context of multi-layered genotype – phenotype maps. For example, the analysis of the glycolytic pathway by Orlenko et al. (2016a, 2016b) shows that the neutral (symmetrical) model yields stasis over long-term evolution, whereas the biased (asymmetrical) model gives shifting patterns of rate-limiting enzymes for pathway flux that is consistent with observations of such shifting patterns across the tree of life. Their introduction of systems biology into the analysis has also been my motivation for developing the general framework presented here for mechanistically linking genotype to phenotype based on a mathematically rigorous definition of biochemical phenotypes (Savageau et al. 2009) and their integration into a space-filling structure in the space of biochemical parameters (Valderrama-Gómez et al. 2020). Another set of problems noted by Liberles is the full reconciliation of observations over short-term vs. long-term evolution, which is an open question. Nevertheless, here the implications of bias mutational processes on phenotype evolution are likely to be of general importance.

The phenotype-centric approach provides a novel theoretical framework to pose and answer questions of phenotype-specific mutation rates and ranking of phenotype frequencies in the population under non-selecting and selecting conditions. The PDS framework makes a key distinction between ‘*entropy increasing/entropy decreasing’* mutations, which cause genetically determined parameter values to change in the direction of an increase/decrease in entropy [see also Stoltzfus (1999)], and ‘*beneficial/detrimental’* mutations, which cause the integrated activities of the entire system to change in the direction of an increase/decrease in phenotype fitness. The two causes are separable. The importance of the distinction can be exemplified by considering the consequence for a population evolving in a temperature gradient (Zhang et al. 2018; Wooliver et al. 2020).

In an idealized case, if the population finds itself in a new environment with a *higher* temperature than the one in which it was previously adapted, the binding of a regulator will now be less effective (higher temperature implies looser binding). The fitness of the organisms will typically decrease. An *entropy-decreasing* mutation causing tighter binding of the regulator can improve fitness. Conversely, an *entropy-increasing* mutation causing an even looser binding can cause a further reduction in fitness. The argument is different if the population finds itself in a new environment that has a *lower* temperature. The binding of the regulator will now be too tight (lower temperature implies stronger binding). The fitness of the organisms will typically decrease. Now, an *entropy-increasing* mutation that causes a looser binding of the regulator can improve fitness. Conversely, an *entropy-decreasing* mutation that causes an even tighter binding can cause a further reduction in fitness. Thus, depending on the environmental condition, an entropically probable mutation at the level of the molecular mechanism can cause either a beneficial or detrimental effect on fitness at the level of the integrated system (Figs. 5 and 9). These same distinctions provide a mechanistic context for interpreting the large differences in frequency of positive-effect mutations that have been discussed by Bondel, et al. (2019).

The PSD framework can distinguish and quantify various phenomena. For example, it distinguishes among three contributions to phenotype-specific mutation rates: phenotype volume [related to the “systemic/organizational” biases of Stoltzfus (1999)], size effect and directional bias, and selection (Fig. 5); it distinguishes among three contributions to the equilibrium distribution with neutral fitness effects (**Eq. **13): mutation alone and selection alone, which nearly balance, and mutation-x-selection (mutations generated specifically by the selected phenotype), which is only significant with extremely strong selection (Fig. 6); it quantifies the different time scales of evolution between equilibria under selecting and non-selecting conditions (Fig. 7).

### Summary of Results Old and New

The findings in **RESULTS** agree with many well-known phenomena in theoretical population genetics. Examples include stronger selection is needed to counteract higher mutation rates, evolution can be faster with higher mutation rates, positive-effect mutations are rare in well adapted systems and small effect mutations are common, and the characteristic distributions observed in *directional* (Darwin 1859; Mitchell-Olds et al. 2007) and *stabilizing* (Charlesworth et al. 1982; Campbell & Reece 2002) selection; *mutation-selection balance* (Barton 2007; Lynch 2010), and cryptic variation under non-selecting conditions (Paaby et al., 2014; Zheng et al. 2019).

However, in all these cases the PDS framework provides a more nuanced understanding of their underlying molecular mechanisms with phenotype-specific mutation playing a role in each. For example, the phenotype distribution with no size effect, directional bias or differences in growth rate under the non-selecting condition, which might be expected to produce a uniform distribution of mutant effects, is weakly directional even though no selection is involved (Fig. 5A**,** Blue); the causal fitness characteristic is the robustness (polytope volume) of phenotypes with phenotype #6 dominating. The phenotype distribution with size effect and directional bias but no differences in growth rate under the non-selecting condition is more strongly directional even though no selection is involved (Fig. 5A**,** Black), with phenotype #6 dominating; the causal fitness characteristics are robustness and entropy. Although the phenotype distribution with size effect, directional bias and protein burden differences in growth rate under the non-selecting condition may also appear to be directional (Fig. 5B**,** Red), with phenotype #11 dominating, it is actually balancing since the causal fitness characteristics are a balance between protein burden differences in growth rate in one direction and entropy in the other. Furthermore, the point of balance is a function of the general mutation rate *m*, which is 10^–7^ in this case. With a higher general mutation rate *m,* the balance shifts in favor of entropy (Fig. 5B**)**, and as *m* approaches 10^–4^, entropy dominates to such an extent that the distribution suggests directional selection. The phenotype distribution with size effect, directional bias and protein burden differences in growth rate under the selecting condition is a more complex balancing selection (Fig. 5C**,** Black), with phenotype #7 dominating; the causal fitness characteristics are a balance between protein burden differences in growth rate in one direction and the selective advantage of oscillation and entropy in the other. The general mutation rate (*m* = 10^–7^ in this case) also plays a causal role in the balance. The distribution of cryptic variation present under non-selecting conditions differs, depending on whether the fitness effects of mutations are neutral (Fig. 5A, Black) vs. near-neutral (Fig. 5B, Red). Although it is difficult to measure such small differences in fitness experimentally, the resulting distributions are markedly different, as are the results under selection (Fig. 9). The causal fitness characteristics involved in the balance are entropy, protein burden differences in growth rate, and genomic mutation rate; the first is dominant in the neutral case, the second is dominant in the near-neutral case, and the third can eliminate the distinction between neutral and near-neutral at sufficiently high rates (Fig. 5B, Blue).

Other results are new, e.g., there is an optimal mutation rate for each phenotype (Figs. 5 and** S7**); the percentage of positive effect mutations is smaller when equilibrium is dominated by phenotypes with high entropy and larger when dominated by those with low entropy (Fig. 9); evolution is slower in the former and faster in the latter; there are many changes in population rank with weak selection (Fig. 7), and few with strong selection (not shown); a non-selected phenotype can increase (without hitch-hiking) as an indirect result of selection for a different phenotype connected by a high phenotype-specific mutation rate (**Fig. S8**); and back calculation of selection coefficients is possible from well-characterized distributions (Fig. 9). We also provide evidence suggesting that experimental evolution in chemostats can be used to experimentally test predictions made possible by the PDS framework (Fig. 8).

We return to the fundamental question raised at the outset and ask, what is the relevant distribution of phenotype frequencies to consider from which there is evolution of new phenotypes? This is still an open question. New phenotypes will grow to dominance when the population suddenly finds itself in a selecting condition because of a change in genotype or a change in environment. The results for the clock model suggest that the equilibrium distribution of the full repertoire in the non-selecting condition with neutral fitness effects, might be the most relevant to consider (Fig. 9). However, even small differences from neutrality that are experimentally undetectable, such as protein burden effects, can result in a marked difference in the distribution (Fig. 5) that argue against its relevance in the case of the natural *lac* operon.

Finally, it should be noted that although we have emphasized qualitatively distinct phenotypes, *quantitative* variants exist within each phenotypic region in Design Space. Thus, the phenotype-centric approach also provides the opportunity to explore finer changes in quantitative characteristics such as frequency, phase and amplidude of the oscillations within the region of phenotype #7 (Lomnitz and Savageau 2013). Such results would be relevant to the work of Ouyang et al. (1998) showning that mutants with small changes in frequency of the cyanobacteria circadian clock experience negative selection when their frequency differs from that of the environmental light–dark cycle.

## Methods

Methods developed in this work are described in the sections DERIVATION OF PHENOTYPE-SPECIFIC MUTATION RATE CONSTANTS and POPULATION DYNAMIC EQUATIONS. Associated computational tools with further details can be accessed through the Design Space Toolbox v.3.0, which is freely available for all major operating systems via Docker. After Docker has been installed, running the following commands in a terminal window will provide access to the software:

1. docker pull savageau/dst3
2. docker run -d -p 8888:8888 savageau/dst3
3. Access the software by opening the address http://localhost:8888/ on any internet browser.

Please refer to Valderrama-Gómez et al. (2020) for detailed installation instructions and troubleshooting. Several iPython notebooks are provided for tutorial purposes and others for reproducing figures in the main text and supplementary information. These notebooks can be found within the Docker image (savageau/dst3).

### Supplementary Information

Below is the link to the electronic supplementary material.

Supplementary file1 (DOCX 1414 KB)
